# Environmentally friendly Fe^3+^-activated near-infrared-emitting phosphors for spectroscopic analysis

**DOI:** 10.1038/s41377-022-00857-x

**Published:** 2022-06-13

**Authors:** Jing Wang

**Affiliations:** Ministry of Education Key Laboratory of Bioinorganic and Synthetic Chemistry, State Key Laboratory of Optoelectronic Materials and Technologies, School of Chemistry, Sun Yat-Sen University, Guangzhou, Guangdong 510275 China

**Keywords:** Optical materials and structures, Inorganic LEDs

## Abstract

Highly efficient Fe^3+^-activated Sr_2-*y*_Ca_*y*_(InSb)_1-*z*_Sn_2*z*_O_6_ broadband near-infrared-emitting phosphors with tunable emission from 885 to 1005 nm are developed as alternative for primarily studed toxic Cr^3+^-activated near-infrared-emitting phosphors for application in spectroscopy analysis.

Near-infrared (NIR) spectroscopy is an emerging technology that offers unparalleled merits in analysis such as fast and non-invasive process. This technique typically works by exploiting characteristic NIR absorption of the chemical compounds containing O–H, C–H, and N–H bonds, which has been widely applied in food processing and agricultural applications^1^. The NIR light sources play an important role in the NIR spectroscopy technology. The emission of the desired NIR light source should be sufficiently broad to provide more information on the functional groups. With the popularity of smart devices and growing public concern of healthy diet, compacted spectrometers need to be combined with portable devices to facilitate daily food analysis^2,3^. Therefore, it is necessary to develop small-sized NIR light sources. Traditional NIR light sources and NIR light-emitting diodes (LEDs) fail to meet the requirements of compactness and broadband emission, respectively. Inspired by the commercial white LEDs, NIR-emitting phosphor-converted LEDs have been developed, which exhibit both small size and tunable broadband emission. However, the development of efficient and broadband NIR-emitting phosphors remains a challenge.

Octahedrally coordinated Cr^3+^ is a primary activator candidate for NIR emission. When it is in a weak crystal field, it can give rise to broadband emission in the range of 650–1200 nm, originating from the spin-allowed ^4^T_2g_ → ^4^A_2g_ transition^4^. Moreover, it can efficiently absorb 460 nm blue light to match the commercial blue LED chips. However, Cr^3+^ is easily to coexist with Cr^4+^ and Cr^6+^ under oxidizing conditions, which limits the NIR luminescence efficiency and increases the chromium toxicity of the phosphors. Thus, these Cr^3+^-activated phosphors are not suitable for some application fields that requires high safety^5^. Very recently, other activators such as Bi^3+^, Eu^2+^, and Mn^2+^ doped NIR-emitting phosphors have also been reported, but their emission is near the deep-red light region^6–8^. For spectroscopic analysis, NIR-emitting phosphors should efficiently emit light in an appropriate NIR spectral range to guarantee good sensitivity. Another activator, Fe^3+^, is an essential metal ion in human body, which can be a candidate activator to address the toxicity issue of chromium^9^. Lin and colleagues have chosen the friendly Fe^3+^ dopant as a potential alternative and developed a series of highly efficient and emission-tunable Fe^3+^-activated A_2_BB’O_6_ (A = Sr^2+^, Ca^2+^; B, B’ = In^3+^, Sb^5+^, Sn^4+^) broadband NIR-emitting phosphors by cation substitution^10^.

The photoluminescence of Fe^3+^ with intraconfigurational d–d transitions has been known and studied for years. The previously reported emission wavelengths of Fe^3+^ activated phosphors are commonly located in the red and far-red light regions^11,12^. In addition, Fe^3+^ is generally considered as a quencher in the field of phosphors because of its spin- and parity-forbidden nature of transition. However, in the work presented here, the authors achieve unprecedented long-wavelength NIR emission of Fe^3+^ by using the perovskite-derived host compound Sr_2-y_Ca_y_(InSb)_1-z_Sn_2z_O_6_. Through adjusting host composition by cation substitution, the crystal field strength around Fe^3+^ is successfully regulated, providing a feasible strategy for manipulating the emission wavelength of this activator. As a result, continuously tunable emission of Fe^3+^ luminescence from 885 to 935 and then to 1005 nm is realized by the designed cation substitution of Ca^2+^ for Sr^2+^ and further cosubstitution of [Sn^4+^–Sn^4+^] for [In^3+^–Sb^5+^]. After Ca^2+^ incorporation, the luminescence efficiency is significantly improved. The obtained Ca_2_InSbO_6_:Fe^3+^ phosphor reaches an ultra-high internal quantum efficiency (IQE) of 87%. Such high IQE occurs with long emission wavelength beyond 900 nm is rare and particularly important for the broadband NIR-emitting phosphors. The application potential of the as-synthesized phosphors in NIR spectroscopy analysis is also demonstrated Fig. 1.

**Fig. 1:**
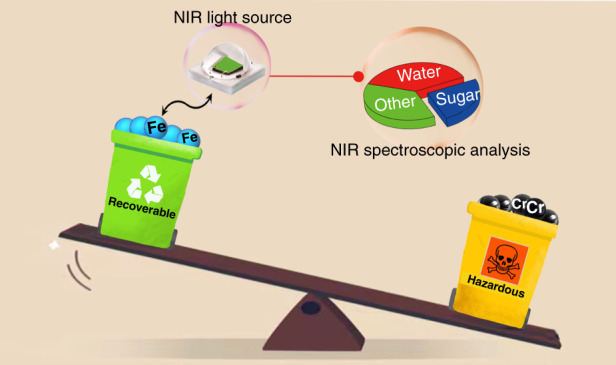
Design principle and application of environmentally friendly NIR phosphor. Schematic view of Fe^3+^-activated near-infrared-emitting phosphors for NIR spectroscopic analysis

Although the development of Cr^3+^-doped NIR-emitting phosphors is the mainstream of current research, other kinds of NIR-emitting phosphors can enrich the types of NIR luminescent materials to meet different application requirements. This presented work by Lin et al. provides new insights into the luminescence of Fe^3+^-activated phosphors and contributes to expanding the family of NIR-emitting phosphors, and promotes the application of NIR-emitting phosphor materials in spectroscopic analysis.
